# Critical Role of Mg^2+^ Ions in RNA Folding Transitions: Anchoring the A‑Minor Twist in the SAM-II Riboswitch

**DOI:** 10.1021/acs.jpcb.5c02586

**Published:** 2025-09-02

**Authors:** Rafael G. Viegas, Anushree Sinha, Avijit Mainan, Karissa Y. Sanbonmatsu, José N. Onuchic, Susmita Roy, Vitor B.P. Leite

**Affiliations:** † Federal Institute of Education, Science and Technology of São Paulo (IFSP), Catanduva, São Paulo 15808-305, Brazil; ‡ Department of Physics, 135131 São Paulo State University (UNESP), Institute of Biosciences, Humanities and Exact Sciences, São José do Rio Preto, São Paulo 15054-000, Brazil; § Department of Chemical Sciences, 99007 Indian Institute of Science Education and Research Kolkata, Mohanpur, West Bengal 741246, India; ∥ Theoretical Biology and Biophysics Group, Theoretical Division, 5112 Los Alamos National Laboratory, Los Alamos, New Mexico 87545, United States; ⊥ New Mexico Consortium, Los Alamos, New Mexico 87544, United States; # Department of Chemistry, 3990 Rice University, Houston, Texas 77005, United States; ∇ Department of Physics and Astronomy, 3990 Rice University, Houston, Texas 77005, United States; ○ Department of Biosciences, 3990 Rice University, Houston, Texas 77005, United States; ◆ Department of Physics and Mathematics, Institute of Chemistry, São Paulo State University (UNESP), Araraquara, São Paulo 14800-060, Brazil

## Abstract

Magnesium ions (Mg^2+^) play a crucial role in stabilizing various RNA tertiary motifs, such as pseudoknots, G-quadruplexes, kissing loops, and A-minor motifs, to name a few. Despite their importance, the precise location and role of Mg^2+^ ions in RNA folding are challenging to characterize both experimentally and computationally. In this study, we employ an all-atom structure-based model integrated with the dynamic counterion condensation (DCC) model to investigate the folding and unfolding transitions of apo SAM-II riboswitch RNA at physiological concentrations of Mg^2+^. Using the Energy Landscape Visualization Method (ELViM), we trace the transitions between conformational phases, focusing on magnesium interactions. ELViM reveals key structural ensembles during the transition from the unfolded to the folded state, facilitated by a partially folded intermediate, which is conformationally similar to that found in early ^13^C-CEST NMR. Interestingly, this study finds the rate-limiting transition from the unfolded state to this intermediate initiated by the formation of an A-minor twist interaction, a stable scaffold in the aptamer domain, stabilized by specific Mg^2+^ coordination. The contact probability map shows that this specific Mg^2+^ bridges a helical region and an internal loop, mitigating electrostatic repulsion at the phosphate level. As a result, a set of hydrogen-bond-mediated interactions between the loop and the minor groove of the helix is stabilized, supporting the formation of the A-minor twist. This study underscores the critical role of Mg^2+^ in driving the rate-limiting event of RNA folding and highlights its strategic location in stabilizing the A-minor twist motif, essential for the global packing and regulatory function of the SAM-II riboswitch aptamer.

## Graphical abstract

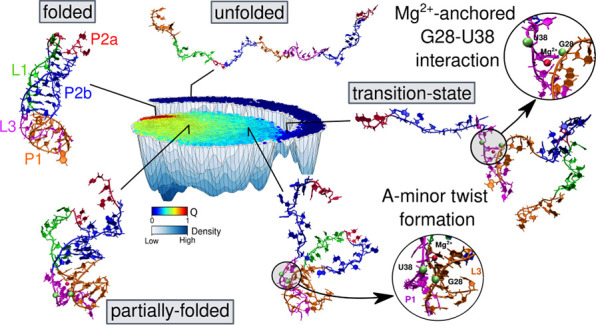

## Introduction

RNA, with its remarkable structural versatility, adopts intricate tertiary folds that are essential for mediating critical molecular interactions within the cell.^1–5^ However, the folding process is inherently challenged by strong electrostatic repulsion between the negatively charged phosphate groups along the RNA backbone.^6,7^ This electrostatic repulsion is largely mitigated by counterions, which help neutralize the backbone charges and facilitate folding, although their efficiency and mechanism of action can vary significantly depending on ion type and local structural context.

A striking example is that millimolar concentrations of Mg^2+^ can stabilize RNA tertiary motifs, such as pseudoknots and kissing loops, which remain weakly stable even under high concentrations of monovalent cations like K^+^, Na^+^.^8–13^ Unlike monovalent cations, which offer only modest stabilization, Mg^2+^ provides a strong electrostatic interaction and can form more robust bridges between phosphate groups. With a high charge density and a small ionic radius, Mg^2+^ effectively neutralizes backbone repulsion and closely interacts with RNA without causing significant structural distortion. This stabilization is crucial for maintaining complex folding patterns and binding sites essential for RNA function.^9^ Despite the significance of Mg^2+^ ions being acknowledged, pinpointing their exact positions and roles in RNA folding presents a formidable challenge.

Like other RNAs, riboswitches also depend on Mg^2+^ for tertiary structure stability.^14–17^ Riboswitches, a unique category of noncoding RNAs, can bind cellular metabolites and regulate gene expression, adopting various complex secondary and tertiary structural folds.^18–23^ A classic example of translational riboswitch is the SAM-II riboswitch (52 nt), which, upon binding SAM (S-Adenosyl methionine), sequesters its Shine–Dalgarno (SD) sequence to repress translation (translation-OFF state); in the absence of ligand, it allows translation (translation-ON). Previously, using various chemical and biophysical methods including NMR and FRET, Haller et al. highlighted the dynamic nature of the unliganded SAM-II riboswitch, in that its stem-loop element becomes engaged in a pseudoknot fold through base-pairing with nucleosides in the 3′ overhang containing the Shine–Dalgarno sequence.^24^ Later, our computer simulations validated by ^13^C CEST NMR,^25^ smFRET,^24^ Small angle X-ray Scattering (SAXS), and size exclusion chromatographic (SEC) data,^26^ showed that apo SAM-II exists in a dynamic equilibrium between partially open and partially closed states, with partial SD sequestration being ligand-dependent. While these studies clarified the ligand’s role in shifting conformational equilibria, the broader question of how the RNA ion atmosphere governs SAM-II RNA folding even to partially closed state remains open.

In a recent review, we highlighted Mg^2+^’s role as outer-sphere ions influencing RNA folding.^27^ Mg^2+^ typically remains hexahydrated in solution but can interact closely with RNA either through direct coordination or via solvent-separated interactions, commonly referred to as outer-sphere coordination.^28–30^ Draper and others have shown that such outer-sphere Mg^2+^ are essential for tertiary fold stabilization.^13^ In fact our Dynamic Counterion Condensation (DCC) model further demonstrated the importance of these interactions in RNA pseudoknots from BWYV, SARS-CoV-2, and flaviviral RNAs.^31–33^

In the current study, we focus on exploring entire folding/unfolding transitions of apo SAM-II RNA—a process vital for maintaining the translation-ON state, using our DCC model integrated structure-based RNA simulation method.^31,34–36^ Simulations began from the crystal structure of SAM-II riboswitch RNA, which forms an H-type pseudoknot^37,38^ with helices P1, P2a, P2b, and loops L1 and L3 (Figure 1A). Tertiary contacts include L1–P2b interactions forming a triple helix at the SAM-binding site (Figure 1B), and A-minor contacts between L3 and P1, critical for structural integrity.^39,40^ In SAM-II, adenines in L3 (A33, A35–37) twist around P1’s minor groove, creating an A-minor twist motif with key contributions from A33 and A37.^37^ To capture ion-driven RNA folding dynamics under physiological Mg^2+^ concentration, DCC model incorporates explicit Mg^2+^ ions. Mimicking experimental salt condition, 100 mM KCl salt buffer is maintained, but KCl is treated implicitly using Generalized Manning counterion condensation (GMCC) theory.^36^ We visualized the conformational landscape using the Energy Landscape Visualization Method (ELViM),^41,42^ which identifies key folding basins and transition states. Previously applied to proteins and RNA tetraloops,^43–48^ ELViM revealed distinct U (unfolded), PF (partially folded), and F (folded) states for SAM-II.

**Figure 1:**
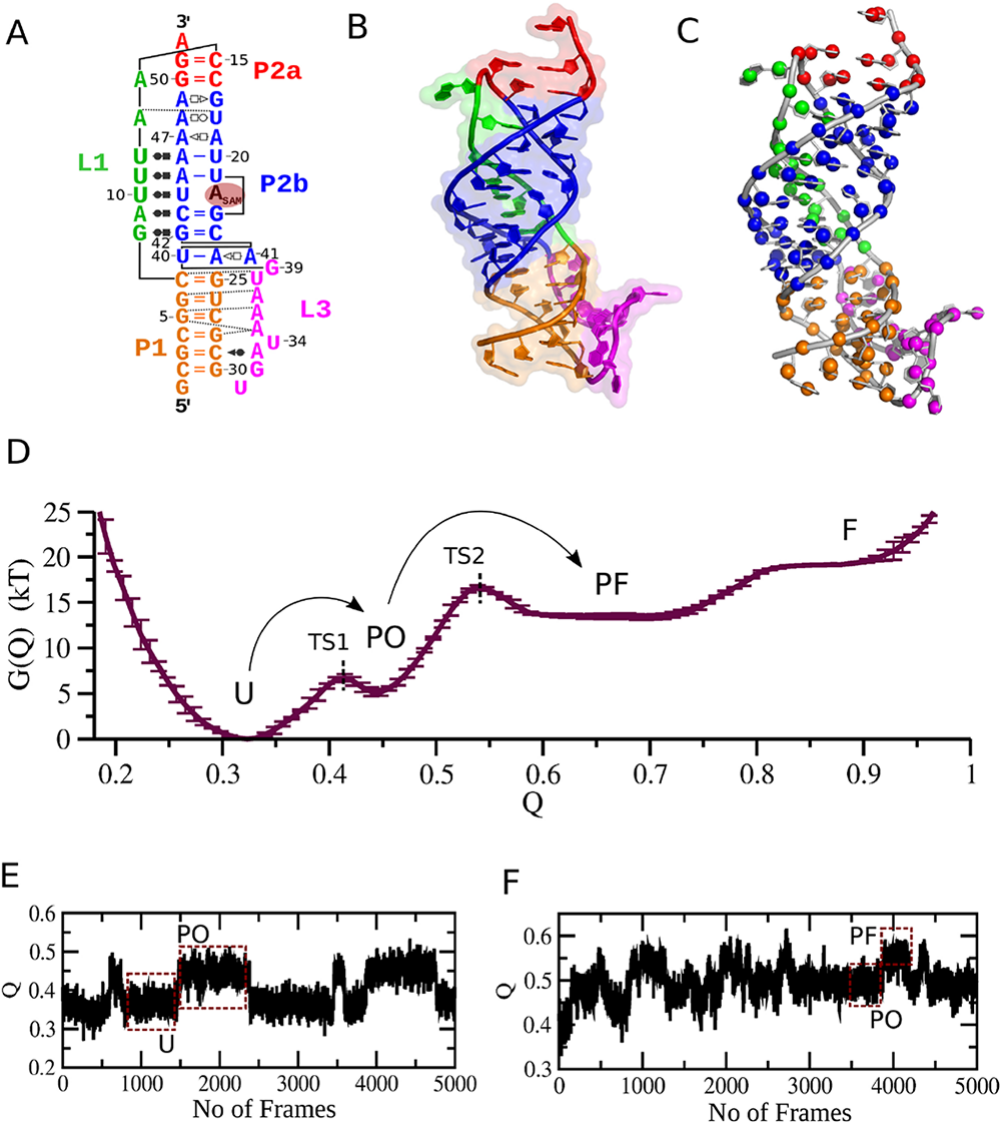
SAM-II riboswitch: structure, free energy profile, and transition dynamics. (A) Secondary structure of SAM-II. The L3 loop interacts with the minor groove of the helix P1 to form the A-minor twist motif. Base-pairings are indicated using the Leontis–Westhof notation,^61^ and dashed lines indicate additional H-bonds. (B) Tertiary structure of the SAM-II riboswitch (PDB: 2QWY, chain A). (C) Coarse-graining scheme used to calculate the dissimilarity, with beads at the center of mass of the phosphate, sugar, and base groups. (D) Free energy profile for SAM-II riboswitch at 101 *T*_R_, [Mg^2+^] = 2.0 mM, identifying unfolded (U), partially open (PO), partially folded (PF), and folded (F) states. (E) Dynamic transition between U and PO states, shown by the evolution of the fraction of native contacts at 91 *T*_R_. (F) Dynamic transition between PO and PF states, shown by the evolution of the fraction of native contacts at 84 *T*_R_.

The study finds that the critical transition from the unfolded (U) to partially folded (PF) state is mediated by the formation of the A-minor twist motif—a rate-limiting step involving P1–P1 and P1–L3 interactions. Mechanistically, it demonstrates how Mg^2+^ ions localize near these structural motifs and stabilize a long-range G28–U38 contact at the motif’s edge. This Mg^2+^-mediated ‘sweet-spot’ interaction reinforces A-minor formation, thereby ensuring the correct folding of the SAM-II riboswitch aptamer essential for its gene-regulatory function.^37^

## Methods

### Atomistic Simulations Using Dynamic Counterion Condensation Model

To explore the structural dynamics and conformational phase space of SAM-II riboswitch RNA, a recently developed dynamic counterion condensation (DCC) model has been employed.^31^ This model combines an implicit-explicit ion environment of monovalent-divalent salts around RNA. It integrates a recently developed Generalized Manning Counterion Condensation (GMCC) theory^36^ into an all-atom Structure-Based Model (SBM) of RNA.^34,49,50^ The DCC model addresses physiological conditions where RNA encounters a mixed monovalent and divalent salt environment. Explicit treatment of Mg^2+^ is essential for governing RNA tertiary packing, while KCl is treated implicitly for computational efficiency using GMCC theory. It is important to note that the explicit treatment of Mg^2+^ ions specifically refers to the solvent-separated outer-sphere Mg^2+^ ions. GMCC theory is an extension of Classical Manning Condensation Theory, which considers counterion condensation in polyelectrolyte systems.^51,52^ Following GMCC, the DCC model incorporates the local charge density of implicit K^+^ ions as a smeared Gaussian shell around each negatively charged phosphate. This Gaussian shell forms the hybrid implicit-explicit interface, excluding the continuum charge density of condensed implicit K^+^ ions around explicit phosphate groups. In the model, K^+^ and Cl^–^ are treated with Gaussian smeared charge, while other ions, including phosphate and Mg^2+^, are treated as point charges for simplicity. The interactions—point charge-point charge, point charge-Gaussian, and Gaussian-Gaussian—are all expressed in terms of Debye–Hückel potential. The complete Hamiltonian used in the DCC model can be written as

$$V=V_{\mathrm{SBM}}+V_{\mathrm{Excl}‐\mathrm{Volume}}+V_{\mathrm{Electrostatic}}$$ 1

where *V*_SBM_ is the all-atom structure-based model potential. The contact level information used in *V*_SBM_ was derived from the crystal structure (PDB: 2QWY) using the shadow algorithm.^53^ *V*_Excl‑Volume_ is the excluded volume effect of explicitly treated hexa-hydrated Mg^2+^ ions, and *V*_Electrostatic_ represents all electrostatic interactions involved in the RNA system. The details of the DCC model and the parameter set used in this model have been extensively discussed in recent literature.^31,32^

### Equilibrium Simulation Details

Atomic coordinates and condensation variables are evolved using Langevin dynamics with a time step of 0.001 τ_R_. We employ underdamped conditions for rapid sampling. For explicit particles, a reduced mass of 1 μ_R_ and a drag coefficient of 1 τ_R_^–1^ were used. Condensation parameters are given a mass of 15 μ_R_ nm^2^ and a drag coefficient of 0.05 τ_R_^–1^ nm^2^. We run 28 production simulations with temperatures varying from 96.5*T*_R_ to 107*T*_R_ to access the whole conformation phase space of the SAM-II riboswitch. Each simulation was run for 200 million time steps. To accommodate Mg^2+^ composition, we created a large cubic box of length 75 nm. The number of Mg^2+^ ions included in the box controls the overall concentration of the corresponding solutes. The excess ions are calculated for the folded and unfolded conformational states with their corresponding representative trajectories, using the cutoff from the analysis of the radial distribution function for Mg^2+^ around the phosphate groups (Table S1 and Figure S1, right). Periodic boundary conditions are applied. Effective charges of −1 are used for each phosphate group and +2 for each Mg^2+^ ion. The parameter set we used for the present simulation is described in the early literature.^31,32^

### Estimation of the Folding Free Energy Profile

To comprehensively explore the conformational landscape of the SAM-II riboswitch, umbrella sampling method^54^ has been employed, using the fraction of native contacts (*Q*) as the reaction coordinate. A series of initial configurations has been generated across discrete *Q* windows by utilizing a slow pulling approach, ensuring Mg^2+^-equilibrated starting structures. For each window, the distribution of explicit Mg^2+^ ions has been reinitialized to improve equilibration, and simulations were extended for 100 million steps per window. To ensure sufficient overlap between adjacent windows, a total of 45 umbrella windows were used along the *Q* coordinate. The free energy was then reconstructed using the Weighted Histogram Analysis Method (WHAM).^55^ Further methodological details of the umbrella sampling protocol are provided in Supporting Information.

### Energy Landscape Visualization Method (ELViM)

ELViM^41,42^ is a versatile multidimensional projection tool used to project effective conformational phase spaces of biomolecules onto a two-dimensional plane. This process involves two key steps: (*i*) calculating a dissimilarity matrix containing structural distances between all pairs of conformations and (*ii*) employing a multidimensional projection technique to generate the low-dimensional representation.

In this study, we estimated dissimilarities using Coarse-Grained (CG) coordinates of the biomolecular system. To achieve this, we employed the Coarse Grain Builder^56^ in VMD^57^ to create three CG beads per nucleotide, positioned at the centers of mass of the phosphate group, the sugar, and the base. A representation of this model is presented in Figure 1C. The dissimilarity between two conformations, denoted as *k* and *l*, is based on *Q*_w_,^58,59^ and defined as follows:

$$q_{w}^{k,l}=\frac{1}{N_{p}}\sum_{i,j\in \mathrm{pairs}}\exp\left[\frac{-(r_{i,j}^{k}-r_{i,j}^{l}{)}^{2}}{2\sigma_{i,j}^{2}}\right]$$ 2

Here, *r*_*i*, *j*_^*k*^ and *r*_*i*, *j*_^*l*^ represent the distances between CG beads *i* and *j* in conformations *k* and *l*, respectively. The weighting parameter σ_*i*,*j*_ varies slightly with the sequence distance and is given by σ_*i*,*j*_ = σ_0_|*n*_b_^*i*^ – *n*_b_^*j*^|^ϵ^, where *n*_b_^*i*^ and *n*_b_^*j*^ denote the residue index to which CG beads *i* and *j* belong. The parameter σ_0_ sets the similarity resolution and was set to 3 Å. *N*_p_ is the total number of CG bead pairs, and ϵ = 0.15.^59^ Dissimilarity, defined as δ_*k*,*l*_ = 1 – *q*_w_^*k*, *l*^, is a unitless measure ranging from zero for identical conformations to one for highly dissimilar ones.

The multidimensional projection is performed using the force scheme algorithm.^60^ Initially, a point representing each conformation is randomly placed in the 2D plane. The algorithm iteratively selects each point as a pivot and slightly adjusts the positions of all other points to optimally approximate the pairwise Euclidean distances (*d*_*k*,*l*_) to the dissimilarities (δ_*k*,*l*_).

To compose the ELViM projection, we subsampled 16 equilibrium trajectories to obtain a converged representation of the conformational phase space. To ensure equilibration, we discarded the first 700 frames of each trajectory and kept frames as follows: one out of every three frames for temperatures sampling the F–PF basin (96.5*T*_*R*_, 97.0*T*_*R*_, 97.5*T*_*R*_, 98.0*T*_*R*_, 98.5 *T*_R_) and the U–PF basin (103.0 *T*_R_, 103.2 *T*_R_, 104.0 *T*_R_, 104.4 *T*_R_, 104.6 *T*_R_); and one out of every seven frames for temperatures sampling the partially folded basin (100.0 *T*_R_, 100.5 *T*_R_, 101.0 *T*_R_, 101.5 *T*_R_, 102.0 *T*_R_, 102.5 *T*_R_). Additionally, 954 frames were taken sequentially during the five U–PF transitions (0.35 < *Q* < 0.65). The resulting ELViM projection is composed of 19,005 conformations.

To illustrate representative conformations from specific regions of the effective phase space, we calculate Local Conformational Signatures (LCS),^42^ determined through the following steps: (i) manually selecting all points within a specific region (e.g., a high-density basin); (ii) computing a matrix of pairwise distance-root-mean-square deviation (dRMSD) values; (iii) identifying the centroid structure that minimizes the average dRMSD; and (iv) displaying the centroid structure alongside some of its nearest neighbors (based on dRMSD) superposed. In this manuscript, the centroid structure is selected and shown as the representative conformation.

## Results

A free energy profile was computed adopting the umbrella sampling technique, revealing four distinct conformational states of the riboswitch—unfolded (U), partially open (PO), partially folded (PF), and fully folded (F) (Figure 1D). Notably, this profile highlights two transition states: (i) transition state 1 (TS1), which delineates the energy barrier between the unfolded and partially open states, and (ii) transition state 2 (TS2), which separates the partially open and partially folded states. A set of 30 simulations was conducted, from which the evolution of the fraction of native contacts reveals the dynamic transitions of the riboswitch between the unfolded (U) and partially open (PO) states, as well as between the partially open (PO) and partially folded (PF) states. Representative trajectories illustrating these breathing motions are shown in Figures 1E,F respectively.

### ELViM Analysis of the Effective Phase Space

In this section, the Energy Landscape Visualization Method (ELViM) is applied to obtain a low-dimensional representation of the conformational landscape of the SAM-II riboswitch, as sampled from the equilibrium trajectories. Supporting Figure S2A also shows the *Q* and RMSD values for each conformation in the ELViM phase space.

Figure 2A presents the ELViM projection colored by the reaction coordinate *Q*. In this effective conformational phase space, each dot represents a sampled conformation, and pairwise Euclidean distances optimally correlate with structural dissimilarity. Thus, nearby dots correspond to structurally similar conformations. The coloring by *Q* highlights the distribution of folded (*Q* > 0.85, deep red), partially folded (0.55 < *Q* < 0.85, light blue to yellow), partially open (0.4 < *Q* < 0.55, dark blue to light blue) and unfolded states (*Q* < 0.4, dark blue). Notably, unfolded conformations cluster in a curved, tail-like region connected to the main projection body through a narrow region that encompasses the transition-state ensemble corresponding to the U–PF transition. Figure S2B, in the Supporting Information, also presents the ELViM projection color-coded based on RMSD values.

**Figure 2:**
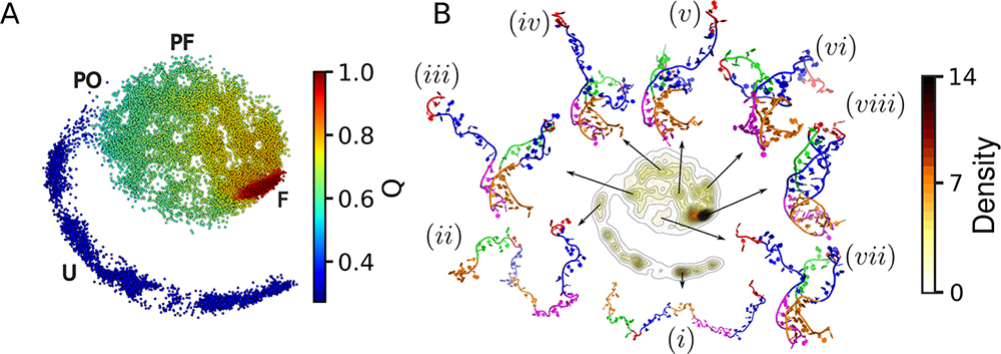
ELViM effective phase-space of the SAM-II riboswitch. (A) Each dot represents a sampled conformation, colored by the reaction coordinate *Q*. Pairwise distances optimally capture structural dissimilarity. Unfolded (U), Partially Open (PO), Partially Folded (PF), and Folded (F) basins are indicated. (B) Density of states estimated using Gaussian KDE. Representative conformations from the densest basins illustrate the conformational landscape.

To gain a deeper understanding of the effective phase space, we estimated the density of data points using a Gaussian kernel density estimate (KDE) and obtained representative conformations (or local signatures) from the densest basins using the Local Conformational Signature (LCS) procedure described in the Methods section. The local signatures are depicted in Figure 2B and provide insight into various regions within the effective phase space. Local signatures (*i*) and (*ii*) correspond to the unfolded basin. While conformations from the basin represented by the local signature (*i*) are completely unfolded, some contacts involving the internal segment of P1 and L3 are eventually observed near the basin characterized by local signature (*ii*).

Transitioning into the PF basin, the representative conformations (*iii*), (*iv*), (*v*), (*vi*), and (*vii*) represent the partially folded state, where base-pairing and stacking interactions in the A-minor twist motif (P1 helix and P1–L3) are established. The comparison of these signatures highlights how the number of base contacts, including local and tertiary pseudoknot interactions, progressively increases, leading to the formation of the P2b helix and the P2b–L1 tertiary structure. Finally, local signature (*viii*) represents the folded state, in which the P2a helix is partially formed. Notably, the P2a helix, which is the last motif to fold, contains the ribosome binding site (RBS), including the Shine–Dalgarno sequence.

For comparison, we also projected the conformational landscape of SAM-II using principal component analysis (PCA). Supporting Figure S3A shows the resulting PCA projection, with dots colored according to their *Q* values. Figure S3B displays the distribution of representative conformations from Figure 2B across both the ELViM and PCA landscapes. The representative conformations exhibit a similar relative distribution in both landscapes. However, the PF and F basins appear more compact in the PCA landscape, while the U–PF transition-state region is more diffuse. This contrast underscores the advantages of the ELViM landscape, which facilitates the identification of the transition-state region and provides a more detailed characterization of the PF basin, including the many local minima populated by partially folded structures.

In our unbiased simulations, five unfolding transitions were sampled, considering sufficiently equilibrated trajectory segments. Supporting Information Figure S4 illustrates the evolution of the *Q* coordinate during these transitions. Additionally, the figure displays the conformations involved in these transitions on the ELViM projection, color-coded according to their time step evolution. Remarkably, all transitions traverse the narrow region connecting the U and PF basins without any jumps, supporting the hypothesis of a well-defined transition-state ensemble.

For a more detailed examination of the transition-state ensemble (TSE), we divided this region in the projection into six sections. Conformations from each section were treated as distinct ensembles, representing different phases of the transition-state ensemble. Representative conformations for each section were obtained as previously described and are shown in Figure 3. Additional conformations are presented in Supporting Information Figure S5, which further illustrates the distribution of the representative conformations across both the ELViM and PCA landscapes. Interaction frequency maps, highlighting well-formed base-pairing and stacking interactions within and between regions, are provided in Supporting Information Figure S6.

**Figure 3:**
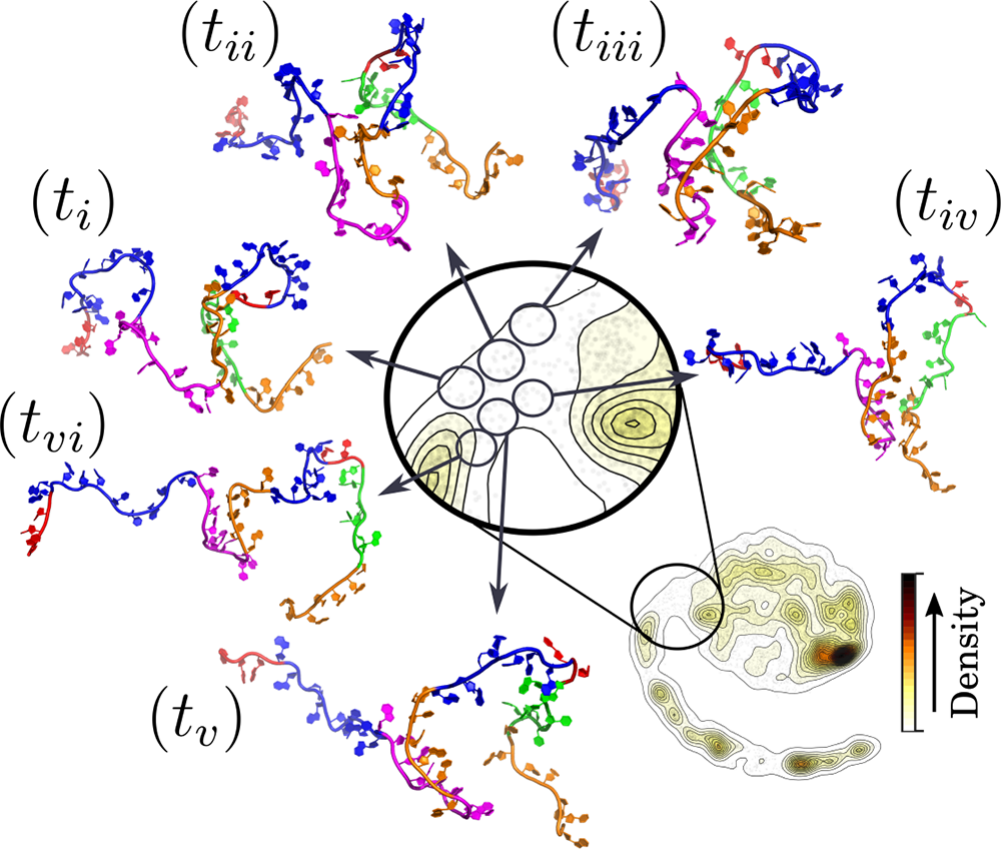
Representative conformations for the transition-state ensemble of the U–PF transition. The transition-state region was arbitrarily divided into six segments, which are illustrated by representative conformations. The analysis of these signatures suggests that the folding process starts with interactions between the internal segment of P1 and the L3, followed by P1–P1 interaction. A more detailed analysis of these regions is provided in Supporting Information Figures S5 and S6.

The representative conformations reveal interactions between the internal segment of P1 and the L3 loop, followed by the formation of the P1 helix and the tertiary P1–L3 interactions. Conformations from the TSE region characterized by signature (*t*_*i*_) (Figure 3) are largely unfolded but bring the L3 loop and the internal segment of P1 into proximity, allowing the initial formation of P1–L3 base-pairing interactions. These interactions become slightly more pronounced in the transition-state ensemble (TSE) regions *t*_*ii*_, *t*_*vi*_, and *t*_*v*_, where the 3′ end remains dynamic and flexible, adopting multiple orientations without forming significant contacts. In this context, the unpaired adenines in the L3 loop begin to exhibit some of the native stacking interactions.

The transition-state ensemble (TSE) regions represented by conformations *t*_*iii*_ and *t*_*iv*_ characterize TS2. The most frequent base pairings in these structures include G28–A35/A36 and C27–A36/A37. These interactions, along with stacking involving unpaired adenines, help stabilize the collapse of the central region. This stabilization facilitates the folding back of the unpaired 5′ end, enabling the formation of base pairings in the P1 helix (G3–C29, C4–G28, G5–C27), culminating in the establishment of the A-minor twist motif.

Lastly, we examined how base pairing and stacking frequencies vary along the reaction coordinate *Q*. The results are presented in Supporting Information Figure S7. This analysis confirms that the initial P1–L3 pairing interactions emerge within the range 0.375 < *Q* < 0.4 and become stronger in the region corresponding to TS1 (0.4 < *Q* < 0.425). A-minor interactions are well established around TS2 (*Q* ∼ 0.5). The pseudoknot interactions between P2b and L1 begin to appear at *Q* > 0.6. Finally, for *Q* > 0.9, we observe the frequent formation of the C16–G50 base pair from the P2a helix, indicating partial formation of this helical region.

### Role of Mg^2+^ in the Rate-Limiting Step of RNA Folding

To investigate the role of Mg^2+^ ions in stabilizing the riboswitch structure, we quantified the average Mg^2+^ population associated with each phosphate group. This was achieved by calculating, for each trajectory frame, the number of Mg^2+^ ions within an 8 Å sphere centered on each phosphate group (P). This cutoff distance between Mg^2+^ and phosphate group has been estimated from the radial distribution function of Mg^2+^ around phosphates (Figure S1). Supporting Information Figure S8 illustrates the average Mg^2+^ population, highlighting that the highest populations occur around residues G6 (P1), C27 (P1), G39 (L3), and A41 (P2b). These key residues are situated within the A-minor twist motif and in proximity to the SAM binding site.

For a dynamic view of the interactions between SAM-II and Mg^2+^ ions, we analyzed how the Mg^2+^ population around each phosphate changes along the reaction coordinate. In the histogram depicted in Figure 4, the first row at the bottom corresponds to the interval *Q* < 0.4 (entirely unfolded) and shows that the Mg^2+^ population is negligible for all residues. As folding progresses, the Mg^2+^ population significantly increases for three groups of residues highlighted in violet. These groups consist of residues G5–C7 and C27–G28 from the P1 helix, and residues A37–G39 from the L3 loop, which form the A-minor twist motif and are involved in the stabilization of the U–PF transition, as previously described. The increased population of Mg^2+^ ions around these phosphates may play a critical role, promoting early collapse by screening electrostatic repulsion and further stabilizing interactions within the A-minor twist motif. At *Q* ≈ 0.6, the Mg^2+^ population increases around residues G8 from L1 and U40–A41 from the P2b helix. Finally, in the PF-F transition, at *Q* > 0.8, the Mg^2+^ population rises in three additional regions: residues U12–A14 from L1, G17–U18, and A46–A48 from P2b. These results suggest preferred Mg^2+^ visiting sites surrounding the SAM-binding site, underscoring the importance of Mg^2+^ in promoting stabilization of the tertiary structure.

**Figure 4:**
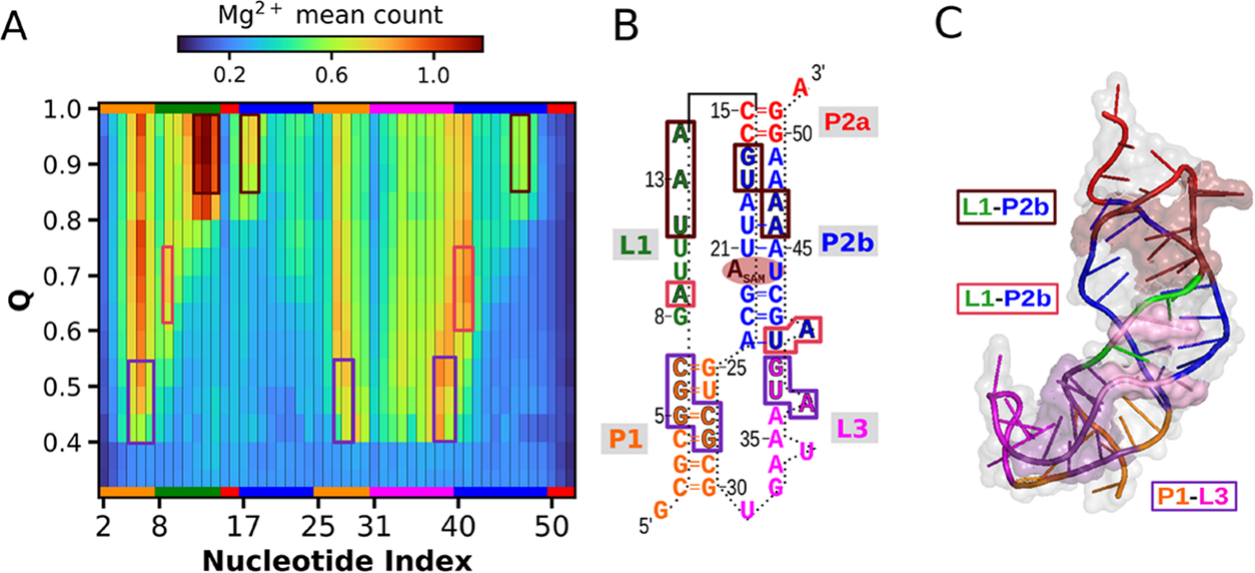
Average Mg^2+^ population per phosphate as a function of *Q* (fraction of native contacts). (A) Total of 140,000 frames were binned by *Q* (bin width = 0.05). For each bin, the average number of Mg^2+^ ions within 8 Å of each phosphate group was calculated. The resulting heatmap shows phosphate indices on the *x*-axis and *Q* bins on the *y*-axis. Residues with the highest average populations are highlighted in the histogram and mapped onto the secondary (B) and tertiary (C) structures with their corresponding color codes.

We also investigated whether specific Mg^2+^-mediated interactions are responsible for the tertiary folding of the structure. To explore this, we calculated Mg^2+^-mediated contact probability map between nonlocal phosphate groups (Figure 5; see Supporting Information for details). Interestingly, we observed a highly frequent long-range interaction mediated by an Mg^2+^ ion between residues G28 and U38. This interaction is the most frequently observed in all simulations sampling the U–PF transition and folded states (from 96.6 T_R_ to 104 T_R_).

**Figure 5:**
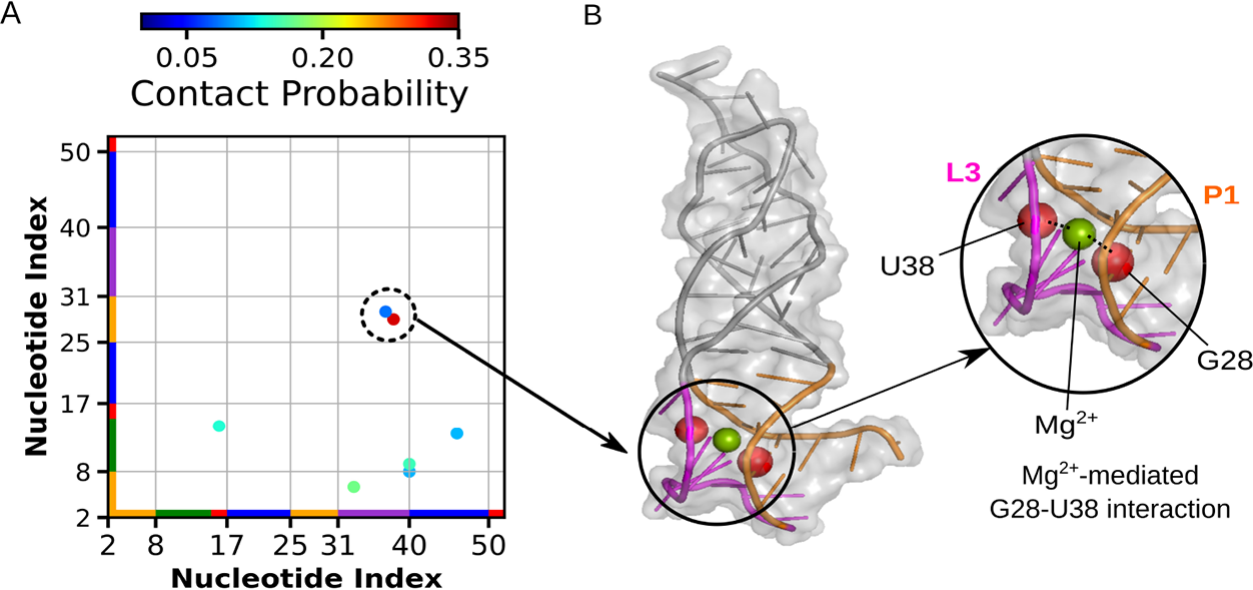
Mg^2+^-mediated contact probability map between nonlocal phosphate groups for the lowest temperature (96.5T_R_). (A) This map illustrates nonlocal contacts between phosphate groups that are mediated by Mg^2+^ ions. Notably, a contact between G28 and U38 is consistently mediated by Mg^2+^ in all simulations sampling the U–PF transition and folded states. (B) Two phosphate groups involved in this contact formation are highlighted as red spheres in the tertiary representation of SAM-II shown on the right.

As previously discussed, these residues—located in the internal strand of P1 and in loop L3—participate in the formation of the A-minor twist motif. Our analysis of base pairing and stacking interactions, both within the folding transition-state ensemble (Figure 3) and across *Q* values (Supporting Information Figure S7), reveals that folding of the SAM-II riboswitch initiates with P1–L3 interactions involving these residues.

Notably, we observed that this key interaction, a rate-limiting step in the folding process,^15^ is mediated by Mg^2+^ ions. Overall, our findings suggest that partially folded conformations are stabilized by a Mg^2+^-mediated contact that anchors A-minor interactions, promotes a preorganized state, and potentially facilitates SAM binding and riboswitch function.

## Discussion

In this study, we explored the folding mechanism of the SAM-II riboswitch. Using molecular dynamics simulations and the Energy Landscape Visualization Method (ELViM), we provided atomistic details of the folding transition process. Our analysis elucidated crucial base-pairings and stacking interactions, as well as the role of Mg^2+^ ions in these processes.

Previous theoretical and experimental studies have suggested that the conformational landscape of the SAM-II riboswitch is characterized by three distinct major basins: unfolded, partially folded, and folded.^15,26,62^ The ELViM projection provided a comprehensive view of the effective phase space, clearly identifying these states. Remarkably, a narrow region connects the U and PF basins via the PO basin, suggesting a specific folding pathway for this transition, involving the formation of the A-minor twist motif.

The PF basin comprises partially folded conformations, where the 3′ end assumes different orientations as the number of base pairings increases toward the F basin. The last part to fold is the P2a helix containing the Shine–Dalgarno sequence. Based on the ELViM projection, we characterized in detail the U–PF transition-state ensemble, providing crucial base-pairing and stacking interactions. These findings were also demonstrated by the free-energy profile for SAM-II RNA representing all of the four conformational states.

In this study, we analyzed five representative unfolding transitions from unbiased simulations, which collectively characterized a single dominant pathway for the U–PO–PF transition. This transition, previously identified as a rate-limiting step,^15^ poses challenges for sampling. Additional breathing simulations, which exhibited reversible transitions along the same route, and the free energy profile obtained from umbrella sampling further supported the existence of this pathway. Moreover, the proposed mechanism is consistent with previous experimental findings. For instance, Haller et al.^62^ proposed, based on NMR and smFRET data analysis, that Mg^2+^ ions stabilize the P1–L3 segment, supporting the formation of the P1 helix and the stabilization of the loop L1 and binding pocket. The complete stabilization of the structure occurs, after SAM binding, with the overall formation of the P2a helix. Chemical probing experiments^37^ have also demonstrated that SAM binding contributes to the stabilization of L1 and P2a/b, while P1–L3 remains mostly unchanged, suggesting that P1–L3 is preorganized in the absence of the ligand. Conversely, Xue et al.^63^ using Replica Exchange Dynamics simulations in the absence of Mg^2+^ predicted an alternative folding pathway, where the folding of P2a/b precedes the formation of the P1 helix. However, to the best of our knowledge, this folding route has not yet been experimentally observed.

We also performed a detailed analysis of the RNA-Mg^2+^ interactions. The importance of Mg^2+^ in stabilizing the tertiary structure of riboswitches has long been addressed by theoretical and experimental studies, indicating that Mg^2+^ is required for SAM-II to achieve its well-folded structure.^25–27,64,65^ In this study, we explored the preferred Mg^2+^ binding sites by analyzing the average number of Mg^2+^ interactions with each phosphate group along the folding process. Our results revealed a cumulative effect of Mg^2+^ coordination that supports conformational changes during the folding process. The average population of Mg^2+^ is initially greater near the P1–L3, supporting the formation of the A-minor twist motif during the U–PF transition. Subsequently, as folding progresses, other preferred sites were identified in the P2b–L1 region. Interestingly, the largest Mg^2+^ average population was observed around U12 and A13 for *Q* values greater than 0.8 (Figure 4). NMR titration experiments conducted by Chen et al.^26^ indicate that the binding of Mg^2+^ ions induces significant perturbation in U12. The authors showed that this perturbation facilitates the formation of new base-pairing, consequently promoting tertiary interactions between loops and stems. This process promotes the transition from the partially folded to a more compact state.

Magnesium-mediated contacts are crucial for the structural stabilization of riboswitches.^26,27^ These ions interact with the phosphate backbone of RNA, creating bridges that hold the complex tertiary structure necessary for the riboswitch’s functionality. This stabilization allows the riboswitch to maintain preorganized conformations that favor ligand binding. In this study, an Mg^2+^ ion was found to mediate a contact between the G28 and U38 residues in all simulations sampling the U–PF transition and folded states, especially captured in the form of TS1 and TS2 ensembles. This Mg^2+^-mediated contact, in conjunction with long-range interactions within the A-minor twist motif, stabilizes a preorganized structure. This stabilization facilitates SAM binding and promotes the folding transition to the closed state.

## Conclusions

In an early review, Doudna and Doherty brilliantly highlighted the contrasts between protein folding and RNA folding.^66^ They noted that, while both the folding processes are challenged by the large configurational entropy required to form ordered native states, each employs unique strategies to achieve its intricate architecture. Despite their differences, the distinctive strategy of RNA folding is still a frontier that remains largely unexplored. Building on this comparison, Chen and Dill later observed that RNA secondary structures often navigate rugged energy landscapes, with complex intermediate states playing a crucial functional role.^67^ Connecting these insights to structure-based mechanisms, we see that in both protein and RNA folding, the burial of hydrophobic residues serves as a driving force. For RNA, this hydrophobic effect of RNA bases is traditionally thought to facilitate the formation of secondary structures, but it also extends to the formation of many base-mediated tertiary structures. A striking example of this is found in the SAM-II riboswitch RNA, where a critical A-minor twist is formed, highlighting the importance of four stacked adenosine residues that stabilize the loop bases against the minor groove. Whether to facilitate the base-mediated or back-mediated tertiary connections, the critical role of ions is always acknowledged to mitigate the backbone-level repulsion.

In this regard, the DCC model has been particularly effective in exploring ion-mediated microscopic phenomena and the rugged energy landscape of RNA, characterizing each folding event of SAM-II riboswitch RNA. This model addresses the computational challenges associated with sampling long-time-scale dynamic processes. Specifically, it treats monovalent K^+^ ions implicitly using the generalized Manning counterion condensation theory^36^ and it is integrated with an all-atom structure-based model of RNA.^15,31,32^ Given that the DCC model has now been validated against numerous experiments,^26,62^ we are extending its applicability by exploring various multiscale phenomena associated with RNA folding and its dynamic ion environment. Additionally, the ELViM method has been employed for the first time to explore the complex multidimensional landscapes of such triplex RNA systems. This method reduces dimensionality and captures a simplified 2D phase space using a purely data-driven approach, without assuming specific reaction coordinates or a reference conformation. This reaction-free approach is particularly useful for capturing the conformational plasticity of RNA under its dynamic ion environment. Using advanced RNA simulation, umbrella sampling approach and energy landscape exploration methodologies, this study provides significant insights into the folding mechanism of the SAM-II riboswitch RNA. We highlight the critical, site-specific role of Mg^2+^ ions in stabilizing its translational ON state in the absence of a ligand. Unlike many other riboswitches, such as SAM-I, where the ON and OFF states represent two alternate folds and a shared sequence is exchanged during the ON–OFF transition,^68^ the SAM-II riboswitch is a unique example. All computational studies and experimental evidence indicate that under physiological Mg^2+^ concentration, it adopts at least a partially folded conformation, which often maintain a dynamic equilibrium between a partially open and partially closed state. These evidence bolster the structural characteristics necessary to facilitate the translational ON-state functionalities of the RNA.

In this direction, our key findings from this study are summarized below:

(i) ELViM analysis has provided detailed insights into the key base interactions that occur during the transition from the unfolded to the folded state, which is mediated by the partially open and the partially folded conformational ensembles.
(ii) This partially folded state represents an open state that culminates in the formation of an A-minor twist motif, a structure consistent with both early experimental data^25,26,62^ and computational findings.^15^ In fact, experiments have shown that this is the predominant conformation in the absence of a ligand, corresponding to the translation-ON state.
(iii) The ELViM projection has also captured the transition state ensemble as the RNA moves from unfolded to partially folded conformations via the partially open state. This suggests that the folding process begins with interactions between the P1 helix and L3 loop, a critical functional motif known as the A-minor twist motif. These findings are well-supported by earlier free energy simulations results, which identified the association of the P1–L3 loop and subsequent formation of the P1 helix as rate-limiting steps in the folding pathway.^15^
(iv) ELViM analysis, along with Mg^2+^-mediated contact mapping, reveals that the formation of the A-minor twist motif is anchored by a Mg^2+^-mediated contact. The influence of Mg^2+^ on the tertiary interaction between the P1 helix and L3 loop was previously assessed and schematically modeled by Haller et al.^62^ However, our study provides a precise structure-based mechanism at an atomistic resolution, showing that Mg^2+^ bridges G28 of the P1 helix and U38 of the L3 loop. This ion-sensitive location for Mg^2+^ is also supported by crystallographic data,^37^ where a Cesium (Cs^+^) ion, rather than Mg^2+^, appears to stabilize this flexible A-minor twist motif.

Overall, the study demonstrates how Mg^2+^-RNA interactions are reorganized throughout the folding process, reducing electrostatic repulsion and facilitating specific base-pairing and stacking interactions. Additionally, it highlights the necessity of advanced atomistic RNA simulation methods and ELViM-like approaches to deepen our understanding of the intricate dynamics involved in ion-mediated RNA folding mechanisms.

## Supplementary Material



## Data Availability

The authors declare that the data supporting the findings of this study are available within the article and its Supporting Information files, or are available from the corresponding authors upon request.

## Notes

Additional methods, supplementary figures, Mg^2+^ radial distribution function, ELViM-PCA comparison, transition ensembles, interaction frequency maps, and a table of excess Mg^2+^ ions across conformational states (PDF)
