# Supplementary material for: Critical Role of Mg2+ Ions in RNA Folding Transitions: Anchoring the A‑Minor Twist in the SAM-II Riboswitch
Source: J Phys Chem B. 2025 Sep 2;129(36):9058–67. doi: 10.1021/acs.jpcb.5c02586 (PMC12434664; doi:10.1021/acs.jpcb.5c02586)
Supplement: Supplementary file 1 [file jp5c02586_si_001.pdf]

Supporting Information: Critical Role of Mg^{2+} ions in RNA folding transitions: anchoring the A-minor twist in the SAM-II Riboswitch.

Rafael G. Viegas,[†,‡,††] Anushree Sinha,[¶,††] Avijit Mainan,[¶] Karissa Y. Sanbonmatsu,[§,‖] José N. Onuchic,[⊥,#,@] Susmita Roy,[*,¶] and Vitor B.P. Leite[*,△,∇]

†Federal Institute of Education, Science and Technology of São Paulo (IFSP), Catanduva, São Paulo, 15808-305, Brazil

‡Department of Physics, São Paulo State University (UNESP), Institute of Biosciences, Humanities and Exact Sciences, São José do Rio Preto, São Paulo, 15054-000, Brazil

¶Department of Chemical Sciences, Indian Institute of Science Education and Research Kolkata, Mohanpur, West Bengal, 741246, India

§Theoretical Biology and Biophysics Group, Theoretical Division, Los Alamos National Laboratory, Los Alamos, New Mexico, 87545, United States

‖New Mexico Consortium, Los Alamos, New Mexico, 87544, United States

⊥Department of Chemistry, Rice University, Houston, Texas, 77005, United States

#Department of Physics and Astronomy, Rice University, Houston, Texas, 77005, United States

@Department of Biosciences, Rice University, Houston, Texas, 77005, United States

△Department of Physics, São Paulo State University (UNESP), Institute of Biosciences, Humanities and Exact Sciences, São José do Rio Preto, SP, 15054-000, Brazil

∇Department of Physics and Mathematics, Institute of Chemistry, São Paulo State University (UNESP), Araraquara, São Paulo, 14800-060, Brazil

††These authors contributed equally to this work and share first authorship

E-mail: susmita.roy@iiserkol.ac.in; vitor.leite@unesp.br

1 Supplementary Methods

1.1 Umbrella Sampling Details

Umbrella sampling[1] is an advanced enhanced sampling technique employed to facilitate the exploration of free energy landscapes characterized by high-energy barriers between distinct conformational states. This method achieves improved sampling efficiency in the barrier regions along a defined reaction coordinate by introducing an external biasing potential, V, commonly referred to as the umbrella potential. In the present study, the fraction of native contacts (Q) is employed as an effective reaction coordinate for estimating the folding free energy landscape of the system. The biasing potential applied along the reaction coordinate is represented as,

$$V = \frac{1}{2}\beta(Q - Q_0)^2 \tag{1}$$

Where β is the force constant and Q_0 denotes the target value of the reaction coordinate, systematically varied between 0 (fully unfolded) to 1086 (folded) to cover the entire conformational landscape (Figure 1D). While the global fraction of native contacts (Q) serves as an effective reaction coordinate for RNA folding studies, conventional contact-counting potentials are inherently discontinuous, often expressed as step functions. However, to ensure compatibility with umbrella sampling, which requires a differentiable potential, a continuous form of the contact-based potential (V_Q) is employed, defined as

$$V_Q = \sum_{\text{contacts}}^{ij} \frac{1}{2}\left(1 - \tanh(\lambda_{ij})\right) \tag{2}$$

where $\lambda_{ij} = \gamma\left(r_{ij} - 1.5\, r_{ij}^0\right)$, with r_{ij} representing the instantaneous distance between atoms i and j, and r_{ij}^0 being the corresponding distance in the native structure obtained from crystallographic structural data (2QWY). This continuous formulation of the contact potential

enables its use as a biasing term in umbrella sampling simulations. The sampling was carried out at a temperature of $101T_R$, to effectively capture the entire folding landscape, including the intermediate states. The error bars depicted in the free energy profile (Figure 1D) represent the standard deviation from the mean, calculated as follows:

$$\sqrt{\frac{\sum_{i=1}^{N}(x_i - \langle x \rangle)^2}{N-1}} \tag{3}$$

1.2 Interaction Frequency Maps

To characterize selected regions of the effective phase space, we calculate interaction frequency maps, which show the average base-pairing and base-stacking interactions. Barnaba[2] was used to annotate well-formed base-pairing and base-stacking interactions. Barnaba is a Python library specifically designed for analyzing nucleic acid structures and trajectories. In our analysis, it was used to annotate the pairing and stacking interactions for each conformation. The interaction frequency map was then computed by averaging the interactions across all conformations within a selected ensemble.

1.3 Mg^{2+}-mediated contact probability map

We utilized contact probability map analysis to evaluate interactions between two non-local phosphate groups mediated by Mg^{2+} ions. This analysis involves two distinct cutoff distances. The first cutoff concerns the distances between the Mg^{2+} ion and the phosphate groups of two residues, denoted as Mg^{2+}-P_i and Mg^{2+}-P_j, where i and j represent the positions of the residues along the phosphate group. Here, a constraint is applied that j is greater than or equal to $i+2$ to maintain the non-local behavior of the phosphate group. These distances are determined based on the radial distribution function (RDF) of Mg^{2+} ions around phosphate groups (Figure S1). Simultaneously, a separate cutoff distance is applied for the distance between two phosphate groups, referred to as P_i-P_j, where two non-local phosphate groups

come into proximity. The specific values for the cutoff distances are taken from RDF analysis, shown in Figure S1.

2 Supplementary Figures

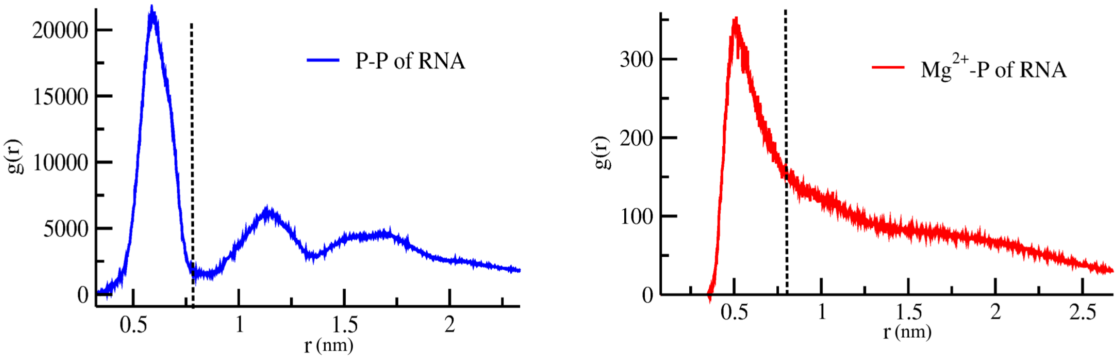

Figure S1: Radial distribution functions (RDF). The RDF of phosphate groups (P-P) is show on the left and of Mg^{2+} ions around phosphate groups is shown on the right. The vertical dashed lines indicate the cutoff value selected for the analysis of the visiting frequency and Mg^{2+}-mediated contacts.

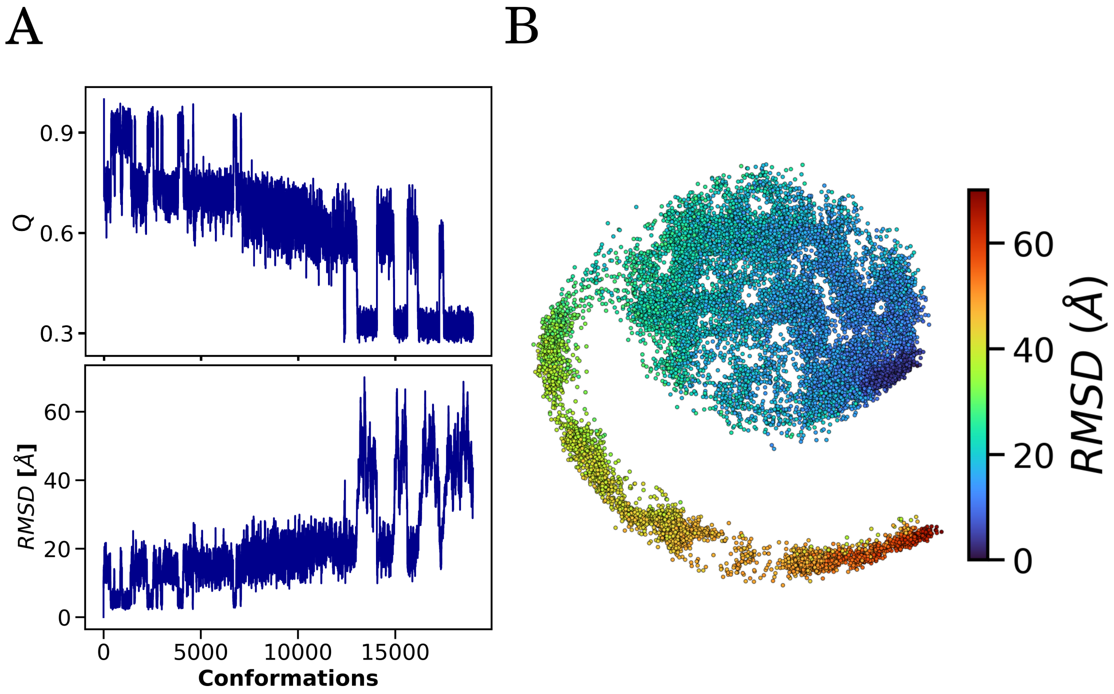

Figure S2: A) RMSD from the crystal structure (PDB:2QWY) for the configurations selected for the ELViM projection. B) ELViM projection with data points colored according to the RMSD from the crystal structure.

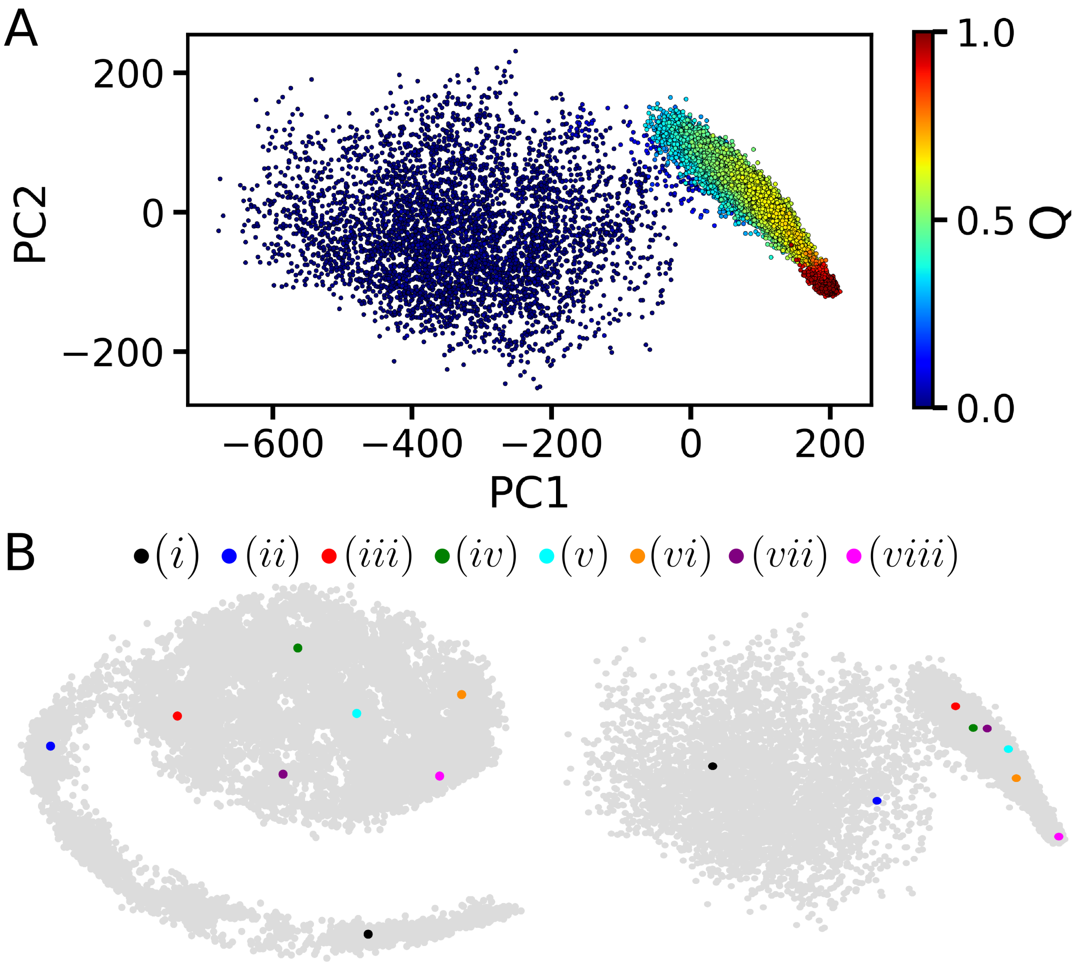

Figure S3: Principal Component Analysis (PCA) landscape. (A) PCA was performed using mass-weighted Cartesian coordinates of all heavy atoms. To remove translation and rotation, structures were centered on the average structure, and this process was refined 10 times until reaching convergence. PCA was performed using the Scikit-learn[3] library. To facilitate comparison with the ELViM landscape, we performed a reflection through the origin and colored each dot based on the fraction of native contacts (Q). The first two components account for 68% of the total variance. (B) The location of the representative conformations shown in Figure 1 of the main manuscript is indicated in both ELViM (left) and PCA (right) landscapes. PCA axes were omitted for better comparison of the landscapes.

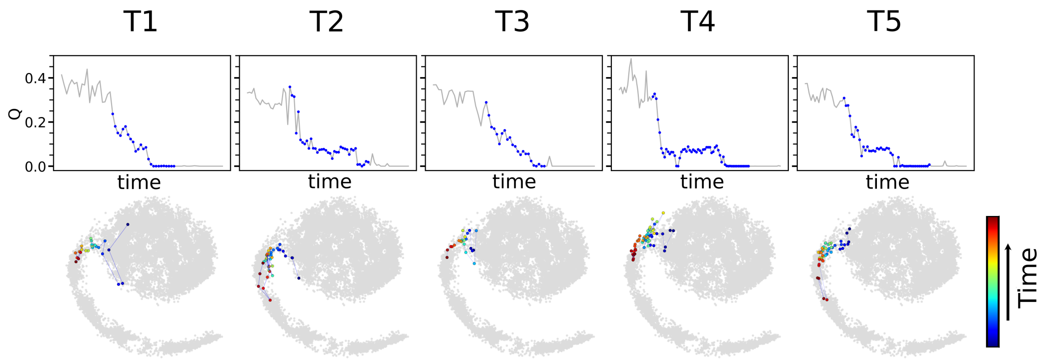

Figure S4: Unfoding transitions. Five transitions from partially-folded to unfolded states were analyzed in this study. The upper panels show the time evolution of the fraction of native contacts along the transition. The lower panels show the time evolution of the transition over the ELViM projection.

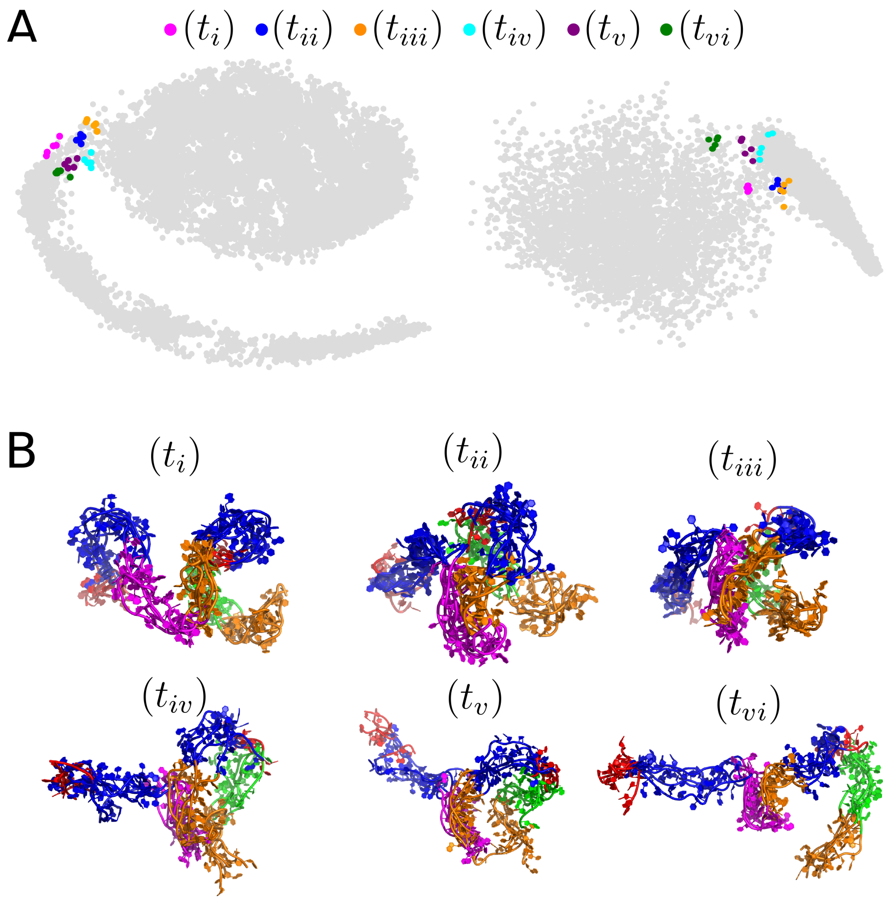

Figure S5: Transition State Ensembles from Partially Folded to Unfolded States. The transition region between partially folded and unfolded states was divided into six segments or transition state ensembles. Using the local conformational signature procedure described in the Methods section, five representative structures were selected from each segment. (A) The position of each representative structure within the transition region is indicated in the ELViM (left) and PCA (right) landscapes. PCA axes are omitted for better comparison of the landscapes. (B) The five representative structures from each segment are superposed to illustrate the structural heterogeneity within each segment.

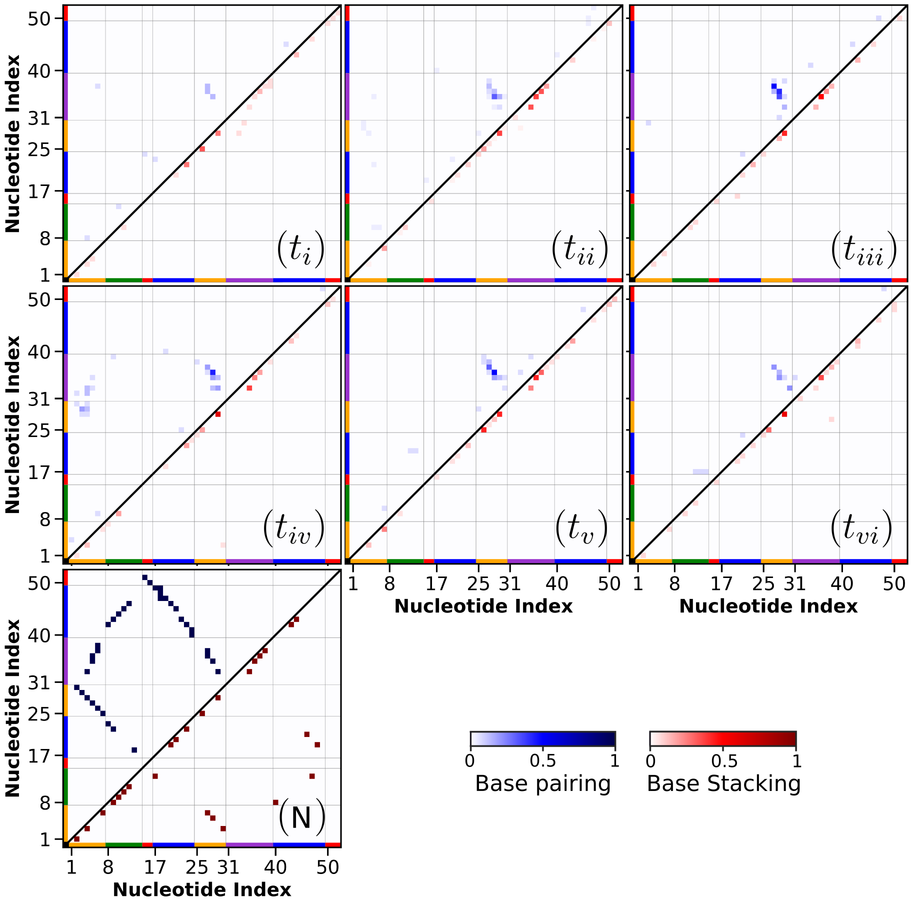

Figure S6: Interaction Frequency Maps for Transition State Ensembles. This figure illustrates the frequency of base pairing and stacking interactions within each transition state ensemble shown in Figure S5. For reference, the interaction map for the crystal structure (N) is also included. Base pairing and stacking interactions were annotated using the Barnaba tool.[2]

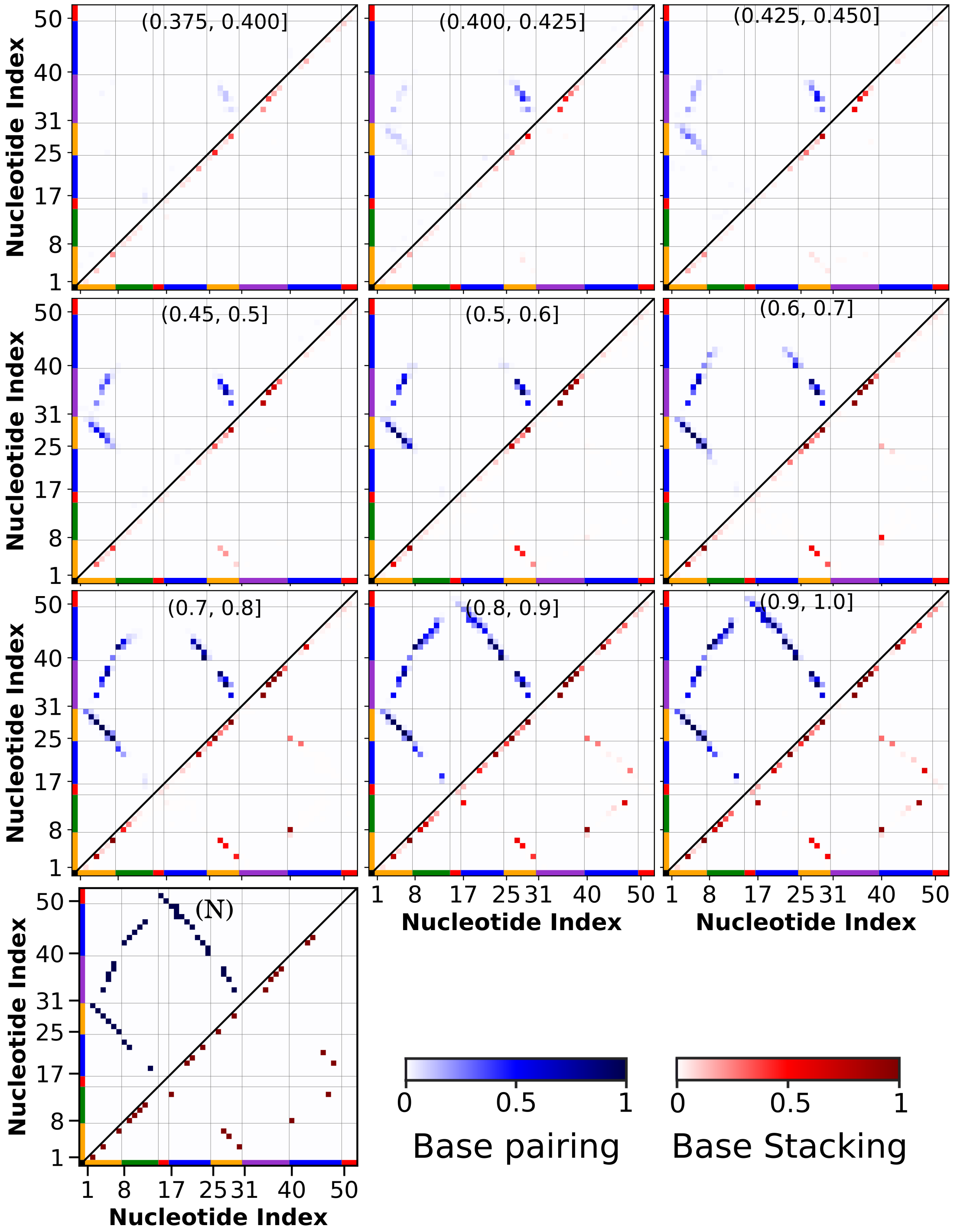

Figure S7: Interaction frequency maps. For this analysis all the 140,000 frames generated in several temperatures were used. Frames were categorized within Q intervals and the interaction frequency were computed within each interval. The interaction map for the crystal structure (N) was also provided for comparison.

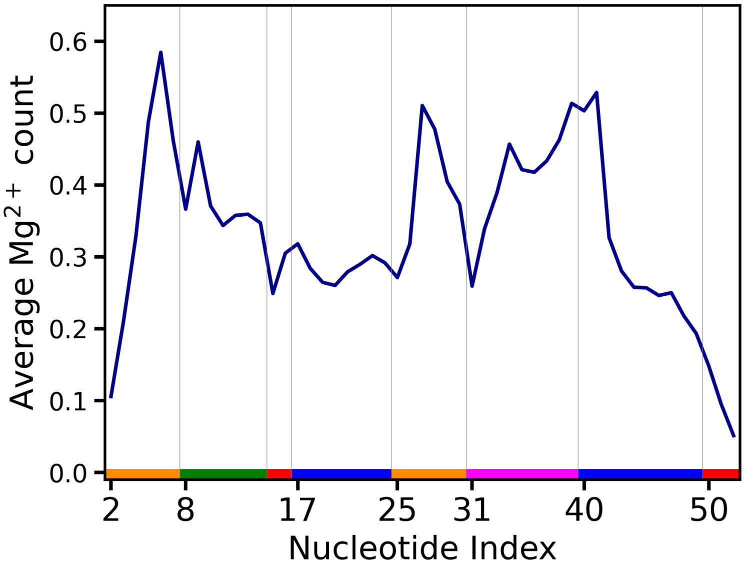

Figure S8: Mg^{2+} visiting frequency. This figures presents the average number of Mg^{2+} ions within a cutoff of 8 Å from each phosphate group. For each phosphate, the average was taken considering all the 140,000 frames.

Table S1: Magnesium ion distribution across conformational states.

Conformational State	Number of Mg^{2+} ions added in 75 nm box	Number of Mg^{2+} in the bulk	Excess ion, $\Gamma_{Mg^{2+}}$
Folded (F)	506	494	12
Unfolded (UF)	506	499	7

References

(1) Torrie, G. M.; Valleau, J. P. Monte Carlo free energy estimates using non-Boltzmann sampling: Application to the sub-critical Lennard-Jones fluid. *Chem. Phys. Lett.* **1974**, *28*, 578–581.

(2) Bottaro, S.; Bussi, G.; Pinamonti, G.; Reißer, S.; Boomsma, W.; Lindorff-Larsen, K. Barnaba: software for analysis of nucleic acid structures and trajectories. *RNA* **2019**, *25*, 219–231.

(3) Pedregosa, F. et al. Scikit-learn: Machine Learning in Python. *J. Mach. Learn. Res.* **2011**, *12*, 2825–2830.
